# Therapeutics

**Published:** 1877-10

**Authors:** 


### II. Therapeutics.

On the State of Therapeutics in Tetanus.—(*Practitioner*, Aug. 1877.) The ancient treatment of tetanus was only palliative, and it was unsuccessful. Modern medicine is armed with a sheaf of weapons which the ancients did not possess; and although yet in despair for a specific, it has so far arrived at such complete alleviation, as often to bring cure with it. A history of the success and failure of the drugs now in use is herein given, in the belief that such a summary makes an appeal to pathology to throw fresh light on the nature of this disease.

Chloroform has had extended trial. Its effects at first sight are most brilliant and satisfactory, nor need it always, in order to relieve spasm, be pushed to narcotism. Great amounts have been used in protracted administration. Simpson narcotized a child for thirteen consecutive days, using ℥100 with success. But the general result is, that while all the fatal symptoms disappear upon the inhalation of chloroform, they return at its removal with unabated violence, and the disease then comes to fatal conclusion without delay. Dr. Panthel, of Limburg, reports experience with chloroform as bearing out this statement. Chloroform is now rarely administered in tetanus.

Chloral has been used in innumerable cases. It is given in large doses, and may be given without fear. A successful case is reported (1875), the daily dose having been 155 grains; and another, a child of twelve and a half years, who took more than 200 grains a day. Dr. Ballantyne, of Dalkeith, gave ℥iij in twenty-four hours, and ℥vjss in three weeks with success. Other and similar cases are well known. Unsuccessful ones were reported in 1874. Alphonse Deu (1875), in a traumatic case, gave as much as 150 grains a day without good result. Chloral was injected into the veins by Dr. Oré (1872), who, though unsuccessful, vaunted his method. It was also tried by Cruveilhier (1874), who also failed, and recommended that a solution, containing one-fifth, in place of one-third, should be used. Lannelongue (1874), using one-fifth, met with same result, and in the *post-mortem* examination found thromboses in the injected veins, and clots in the right heart. Dr. Oré's method was denounced in the Medical Theatre, at Paris (1874), and finally died in the same year before the Surgical Society of Paris, defended by its author to the last. A strength exceeding one-third for subcutaneous injections, injures the skin.

Calabar bean has a high and well deserved reputation. Eibert (1873) pretends that of opium, chloroform, chloral, curare, and Calabar bean, the latter is the only one that acts satisfactorily on the spinal cord. He recommends either previous narcotism by chloral, or the simultaneous administration of atropine, so that both of these combinations have probably been tried. Holthouse (1864) reported two cases, one successful. He used three grains of the extract every two hours, and once four and a half grains in one dose. Maunder added two unsuccessful cases. Dr. Eeben Watson (1867) reported two traumatic cases which recovered. He had given, by accident, nine grains in as many hours. Later he reported six of eight cases successful.

Mr. Ashdown (1868) reported a case in which the drug seemed to fail; and Professor Spence (1875) reported another unsuccessful case, in which a boy of eighteen took twenty-two grains of the extract in three and a half days, and died on the fourth day. Dr. Dickenson (1876) lost a case after having administered subcutaneously seven grains of the extract in the course of an afternoon, without cessation of the spasm being procured. He reports also two successful cases, one of an idiopathic and the other a protracted nature. The drug has the effect of controlling the spasms, and lowering the temperature. If withheld, the paroxysms return ; if pressed, it is dangerous. It is recommended to be used hypodermically in doses of not less than one-third grain of the extract every two hours. It is thought by some to dilate the pupil at first, also, as will curare, to produce spasm.

Opium, combined with chloral, has been successful. Delsol (1874) reports three cases out of four saved by this plan ; but in most cases, where this combination has been used, the chloral received more than half the merit of the cure. Alcoholic cases have been successfully treated by opium alone.

Nitrite of amyl has been tried and failed.

Bromide of potassium, combined with chloral, was used successfully by Drs. Panthel and Carruthers (1874); but the chloral was given in large doses. Bromide of potassium alone has had doubtful success.

Conium has had no flattering result. The cases in which it has seemed successful have been slight ones. Of aconite, some very remarkable results have been given. It was first used in 1846 by Mr. Page. The case was traumatic, and there was a remission of all symptoms after a three minim dose, then recurrence of symptoms, and with the repetition of the medicine, chilliness, cold skin, and clammy sweat, pulse 120, weak and intermittent. Antidotes used were wine and opium. The effects of the drug constitue in themselves a new danger to be met. Curare was first used in Italy by Vella in 1859. It was unsuccessful in his hands. But Cassaignac, in the same year, used it successfully. Its use, however, has passed into disrepute. Belladonna has met with some success. Benoit (1860) failed with it. On the other hand, Peschaux, in the same year, reported a successful case to the Surgical Society of Paris, having injected a solution of $\frac{1}{100}$ of atropine into different parts of the body. Fournier (1860) met with similar success. Strychnia increases, as one would suppose, the symptoms of tetanus. For cannabis Indica no special action has been made out. It has been tried, and failed.

Neurotomy, tenotomy, and amputation are beyond the scope of this paper.

For cold applications to the spine, sufficient material is wanting.

Carpenter (1860) states (reference not verified) that he cured sixteen out of seventeen cases by this plan. The ether-spray to the back has been reported of use.

It is to be hoped that some combination of these agents may be indicated, which will perform what each one of them singly has been found unable to accomplish.

VIBURNUM PRUNIFOLIUM (*Black Haw*).—E. W. Jenks, M. D. (*Gynæcological Transactions*, 1876.) This remedy, used by the writer almost daily for several years, warrants him in speakitg confidently in regard to results obtained from its use. Its most frequent use has been as a prophylactic against abortion. Of course the remedy is worthless when the abortion has already begun by detachment of the ovum. Where the habit of abortion has been formed, the viburnum may be given in the form of the fluid extract from a half teaspoonful to a teaspoonful, four times a day, beginning two days before the regular menstrual date, and continuing it two days longer than the usual menstrual flow. In dysmenorrhœa with profuse menstruation and pain, except when the pain is due to stenosis or mechanical destruction, viburnum affords the patient great relief. The remedy should be given for several days in advance of the period as well as during the time of the flow.

In spasmodic or neuralgic dysmenorrhœa, it is not sufficient alone to give relief, but may be given with advantage combined with sedatives and antispasmodic remedies, such as cannabis Indica, camphor, hyoscyamus, and conium. In that form of dysmenorrhœa with menorrhagia, caused by fibroid growths, it has been given in combination with ergot, with gratifying results. The writer would designate viburnum prunifolium as a uterine sedative, whose action is as pronounced as is that of ergot in causing uterine contraction.

The form of the viburnum used is the fluid extract made from the bark of the root and bark of young shrubs, and newly grown twigs. The dose is a half a drachm to a drachm, repeated every two to six hours.

A NEW TEST FOR BILE-PIGMENT.—Dr. Waller G. Smith. —(*London Lancet*, July).—In a recent paper on the value of tincture of iodine as a test for bile-pigment in the urine, Dr. S. asserts that the value and delicacy of the nitric acid test is not so great as is desirable, and claims for tincture of iodine several advantages. It is easily procured, is not corrosive, the color produced is definite and persistent, and for delicacy it equals nitric acid. The idea is not new, it having been published in a French journal several years ago. The best method of procedure is to place about a drachm of the urine in a test-tube, and then to allow one or two drops of tincture of iodine (B. P.) to trickle down the side of the tube, held nearly horizontally, so that the two fluids may touch, but not mix. If bile pigment be present, a fine green color will almost immediately be developed below the red layer of iodine tincture.

When the test-tube is held against a white surface, three zones of color are distinctly seen; viz., the red iodine layer, the yellow stratum of urine, and the green color between the two. The test succeeds better by floatation than by mixing the fluids, and the green color will persist sometimes for days. If the urine is very dark in color, it should be first diluted with water.

Heat speedily changes the color from green to brown.

Fetid Feet.—As a remedy for this noisome affection Dr. Rumbold recommends bathing the feet in warm water for fifteen minutes just before going to bed. The water should be kept as warm as can be borne, by the addition at intervals of boiling hot water. After the feet are dried and thoroughly rubbed with a coarse towel, an ointment composed of salicylic acid and bromide of potassium, each five grains to the ounce of vaseline, should be applied with considerable friction. Then the feet should be covered with a pair of cotton stockings well warmed.

In an article in the Revue de Thérapeutique, it is stated that an immediate remedy is found in washing the feet with a solution (1 in 100) of chloral and keeping them enveloped in compresses wetted with the same solution. Results as satisfactory, Dr. Burdon has claimed to have been obtained by the employment of a solution (commencing with 3 in 1,000) of the permanganate of potash. Dr. Berthold also indicates an efficacious method which is less troublesome than that of bathing with solutions. It consists in powdering the interior of the patient's socks with a powder composed of one part of salicylic acid and five of starch. This is, too, an excellent mode of treating the local sweating which in fat persons takes place between the scrotum and the thighs, and if not arrested leads to a troublesome eczema and its accompanying pruritus.

